# Effect of Klotho protein during porcine oocyte maturation via Wnt signaling

**DOI:** 10.18632/aging.104002

**Published:** 2020-11-16

**Authors:** Eui Hyun Kim, Anukul Taweechaipaisankul, Muhammad Rosyid Ridlo, Geon A Kim, Byeong Chun Lee

**Affiliations:** 1 Department of Theriogenology and Biotechnology, Research Institute for Veterinary Science, College of Veterinary Medicine, Seoul National University, Seoul, Republic of Korea; 2 Department of Biomedical Laboratory Science, School of Medicine, Eulji University, Jung-gu, Daejeon, Republic of Korea; 3 Department of Bioresource Technology and Veterinary, Vocational College, Universitas Gadjah Mada, Yogyakarta, Indonesia

**Keywords:** Klotho, maturation, oocyte, porcine, Wnt signaling

## Abstract

Klotho protein is well-known as an anti-aging agent, however, several studies have suggested that Klotho protein also increases antioxidant activity and the reproductive system, as Klotho protein is closely associated with Wnt signaling. The objective of our study was to investigate the enhancement of porcine oocyte *in vitro* maturation *via* the Klotho protein-Wnt signaling pathway. Following immunohistochemistry and ELISA, we treated cells with Klotho protein during *in vitro* maturation. Lithium Chloride, a specific activator of Wnt signaling, was subsequently co-administered with Klotho protein. Mature oocytes subjected to treatments were used for the analysis of embryonic development, qRT-PCR, and immunocytochemistry. Treatment with 5pg/ml Klotho protein significantly increased cumulus cell expansion, blastocyst formation rates, and the total cell number of blastocysts. During cotreatment with 5mM Lithium Chloride and 5pg/ml Klotho protein, blastocyst formation rates were the highest in Klotho protein-treated oocytes and the lowest in Lithium Chloride-treated oocytes. Expression levels of Wnt signaling-related transcripts and proteins were significantly impacted by Klotho protein and Lithium Chloride. Moreover, cellular ATP levels and antioxidant activities were enhanced by Klotho protein treatment. These findings suggest a significant involvement of the Klotho protein-Wnt signaling mechanism in porcine oocyte maturation.

## INTRODUCTION

The function of the *Klotho* gene was first discovered in 1997, when a *Klotho*-mutated mouse showed an extreme decrease in lifespan [1]. *Klotho* mutations have since been identified as causing numerous symptoms, including hypogonadism, neurodegeneration, and hearing loss [2]. Additionally, Klotho is found to upregulate cell-surface abundance of calcium channel protein in human kidney cells [3], enhances cognitive ability of mouse brain [4], and suppress autophosphorylation of IGF receptors, which is associated with tumor progression [5]. However, *Klotho* has alternately been suggested to be involved in porcine *in vitro* production (IVP). Lee et al. found that overexpression of *Klotho* in porcine fibroblasts significantly improved the development of cloned porcine embryos [6]. Moreover, several studies have also suggested that Klotho is involved in antioxidant mechanisms. One study demonstrated that the Nrf2-mediated antioxidant mechanism can be induced by the Klotho hormone [7]. Likewise, in the state of oxidative stress, Klotho protein (KP) downregulates PI3K/AKT signaling and enhances the expression of manganese superoxide dismutase for antioxidation [8].

Wnt signaling is known to be involved in various pathological and physiological mechanisms in both invertebrates and vertebrates, especially in carcinogenesis and embryonic development [9–11]. Moreover, several studies have demonstrated the roles of Wnt signaling in porcine oocytes. Unlike the bovine oocytes [12], and similarly to the mouse oocytes [13], inhibition of Wnt signaling shows positive effects on porcine IVM and embryonic development. Conversely, activation of Wnt signaling through specific inhibitors impairs the quality of porcine oocytes and embryos [14, 15]. Lithium Chloride (LiCl) is one of the effective activators of Wnt signaling to reduce estradiol concentration in mouse follicles and significantly impacts mammalian reproduction [16]. Furthermore, overexpression of β-catenin, the main downstream target of Wnt signaling, significantly reduced the expansion of cumulus-oocyte complexes (COCs) in mice [17].

Interestingly, a link between Klotho and Wnt signaling was suggested in a study of human carcinoma cells. Artificial overexpression of Klotho inhibited Wnt signaling, and this was confirmed by reduced β-catenin expression and reduced translocation of β-catenin into the nucleus [18]. Other studies have also shown that Klotho inhibits Wnt signaling in pigs and mice [19, 20]. Therefore, in accordance with these findings, we propose the hypothesis that Wnt signaling inhibition with Klotho may be significant in porcine oocyte maturation.

## RESULTS

### Verification of presence of KP in porcine ovaries and COCs

**Figure 1:**
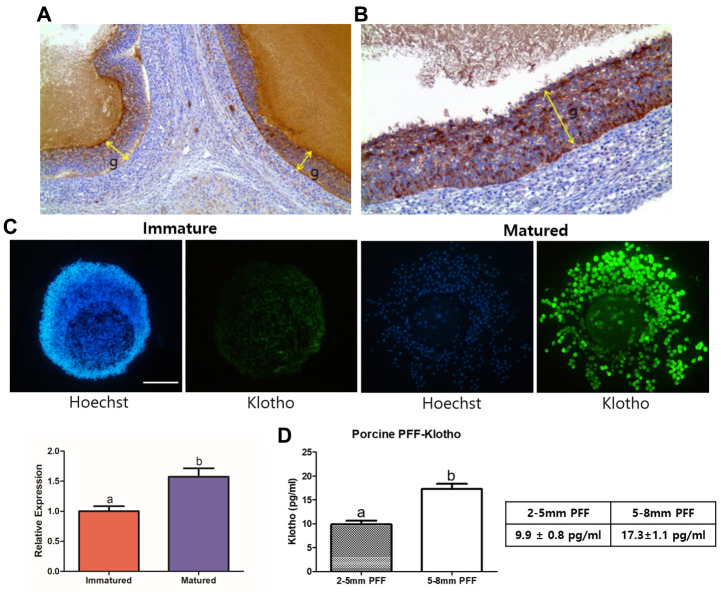
**Validation of the presence of Klotho in porcine ovaries, PFF, and COCs.** (**A**) Results of immunohistochemistry performed on the porcine ovaries. Note the strong positive granulosa cells (g, indicated with arrows) at X100 magnification and (**B**) X200 magnification. (**C**) KP expression in porcine oocytes, assessed using immunocytochemistry. KP is expressed significantly more strongly in mature than in immature COCs (*P* < 0.05). More than 25 COCs from three biological replications were used. (**D**) Results from ELISA of Porcine follicular fluid (PFF) obtained from 2-5 mm- and 5-8 mm-sized follicles. PFF from 5-8 mm-sized follicles showed a significantly higher KP concentration than did that from 2-5 mm-sized follicles. Bars with letters indicate significant differences (*P* < 0.05).

Prior to the start of this study, we confirmed the KP expression in the porcine reproductive system, and particularly in the ovaries and COCs. As shown in Figure 1A and 1B, we found a strong expression of KP in porcine ovaries through immunohistochemistry, and KP expression in mature COCs was also significantly higher than that in immature COCs (P < 0.05) (Figure 1C). This indicates that KP is not only present in COCs, but is also expressed more strongly when the COCs are mature. Additionally, we checked the KP concentration in the porcine follicular fluid (PFF) by ELISA (Figure 1D). PFF collected from 5–8 mm-sized follicles contained a significantly higher KP concentration compared with the PFF of 2-5 mm-sized follicles (17.3 ± 1.1 pg/ml vs. 9.9 ± 0.8 pg/ml, P < 0.05). In accordance with ELISA results, we examined the concentration range of KP treatments for subsequent *in vitro* experiments at 0, 2, 5, and 10 pg/ml.

### KP and LiCl optimization during porcine IVM

**Figure 2:**
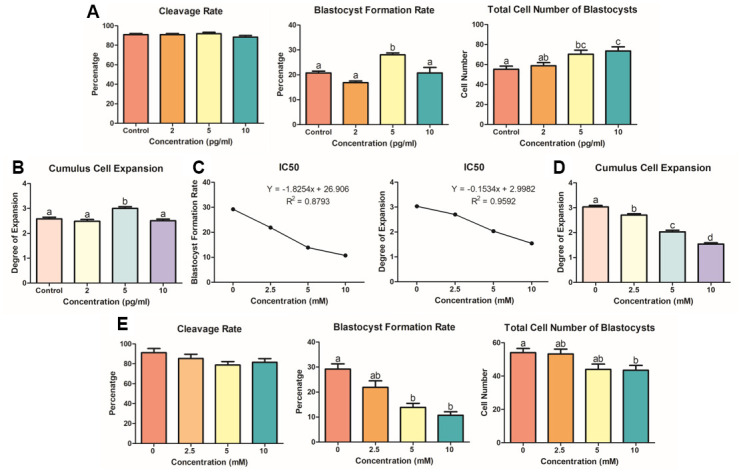
**KP and LiCl optimization during porcine IVM.** (**A**) Treatment with 5 pg/ml KP resulted in the highest blastocyst formation rate in PA-derived embryos, and treatment with 5 and 10 pg/ml significantly increased the total cell number of blastocysts compared to that in the control group. At least 160 oocytes from six biological replications were used. (**B**) Degrees of expansion of CCs in dose-dependent manner of KP. Only the 5 pg/ml KP treatment significantly increased CC expansion. Nine replications of this experiment were performed. (**C**) To specify the optimal concentration for treatment, the IC50 linear equation was applied using values derived from the blastocyst formation and cumulus cell expansion rates. The IC50 values for the optimal concentration were 6.5 mM and 5.3 mM, respectively. (**D**) Cumulus cell expansion of LiCl dose-dependent manner significantly decreased from 2.5 mM. Five replicates were performed. (**E**) Oocytes were treated with LiCl during IVM and used for evaluation of embryonic development. Treatment with 5 and 10 mM LiCl resulted in a significant decrease in blastocyst formation rate. More than 125 oocytes from four biological replications were used. Bars with letters indicate significant differences (*P* < 0.05).

**Figure 3:**
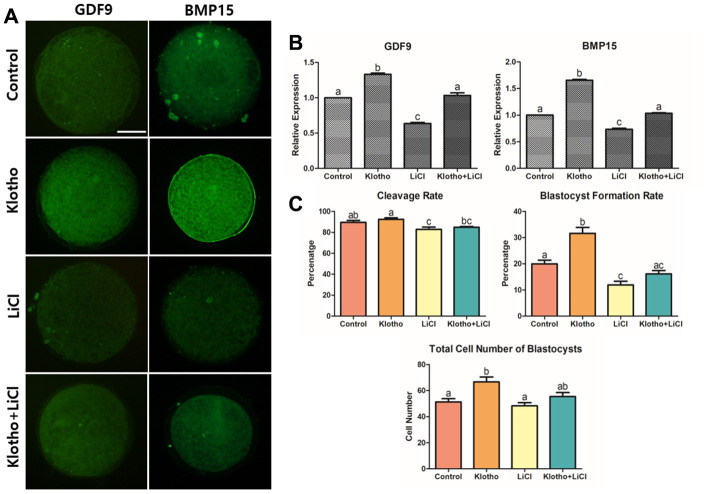
**Co-treatment of KP and LiCl during IVM and their effects on oocyte competence.** (**A**) Expression levels of GDF9 and BMP15 in mature porcine oocytes. More than 20 oocytes from each group were used for the staining. X400 Magnification. (**B**) Treatment with KP significantly increased the competence of the oocytes, while treatment with LiCl decreased the competence. (**C**) Co-treatment of KP and LiCl. KP and LiCl had significant inverse effects on the cleavage rate and blastocyst formation rate compared to those observed in the control group, and the KP-treated group had the highest total number of blastocysts among all groups. A total of six replicates were performed. Bars with letters indicate significant differences (*P* < 0.05).

Optimization of KP and LiCl is necessary to visualize the involvement of these molecules in Wnt signaling. We treated porcine oocytes with KP during the entire process of IVM, using the concentration range that we set through the first experiment (Figure 1). Figure 2A and 2B show the effect of KP treatment on the subsequent embryonic development of the porcine oocytes, as well as its effect on the total cell number of blastocysts and cumulus cell expansion. Oocytes treated with 5 pg/ml KP showed the highest cumulus cell expansion rate compared to those treated with 0 (control), 2, and 10 pg/ml (Degree 3.00 vs. 2.58, 2.48, and 2.50, respectively, P < 0.05). The oocytes treated with 5 pg/ml KP also showed an increased blastocyst formation rate compared to those treated with 0 (control), 2, and 10 pg/ml (28.04 ± 0.77 vs. 20.77 ± 0.75, 16.88 ± 0.63, and 20.77 ± 2.17, respectively, P < 0.05). In terms of the total cell number of blastocysts, oocytes treated with 5 and 10 pg/ml KP showed significantly higher cell numbers than control oocytes (70.38 ± 3.99 and 73.50 ± 4.09 vs. 55.27 ± 3.08, respectively, P < 0.05). However, the cleavage rates showed no differences among the experimental groups. We then examined different concentrations of LiCl (0, 2.5, 5, and 10 nM), as an activator of Wnt signaling in porcine IVM. Firstly, we evaluated cumulus cell expansion in COCs after treatment with LiCl. Results showed a dose-dependent manner in reducing cumulus cell expansion after treatment with LiCl (Figure 2D). Regarding embryonic development (Figure 2E), oocytes treated with 5 and 10 mM LiCl showed a significant decrease in blastocyst formation rate compared to control oocytes (13.88 ± 1.59 and 10.72 ± 1.34 vs. 29.21 ± 2.08, respectively, P < 0.05), and those treated with 10 mM LiCl exhibited a significantly reduced total cell number of blastocysts compared to the control group (43.47 ± 2.89 vs. 54.06 ± 2.45, P < 0.05). However, there were no differences in cleavage rate among the treatment groups. Lastly, we measured IC50 using the values derived from the blastocyst formation rate and total cell number of blastocysts (Figure 2C). We found that 5 mM LiCl was the optimal dose for treatment, as the suggested IC50 values from the blastocyst formation rate and cumulus cell expansion were 5.322 and 6.507 mM, respectively. Each value was calculated using the linear equations depicted in Figure 3B. Therefore, we chose 5 mM LiCl as the optimal treatment dose for subsequent experiments.

### Impacts of co-treatment with KP and LiCl on the oocyte competence

Following optimization of KP and LiCl concentration, their individual and combined effects during IVM on the subsequent embryonic development were analyzed. We measured the expression levels of GDF9 and BMP15, which are representative markers for oocyte competence, in porcine oocytes using ICC. As shown in Figure 3A, both factors showed similar expression patterns. The KP-treated group showed significantly higher expression levels of both GDF9 and BMP15 compared to the control group. In contrast, the LiCl-treated group and the co-treated group showed the lowest expression levels among the groups. Furthermore, the expression levels of both GDF9 and BMP15 in the co-treated group were similar to those in the control (Figure 3B). Figure 3C shows the results of KP and LiCl co-treatment during IVM. The LiCl and co-treated groups showed a significant decrease in cleavage rate compared to the KP-treated group (82.90 ± 2.21 and 84.84 ± 0.77 vs. 92.44 ± 1.40, respectively, P < 0.05). The KP-treated group showed the highest blastocyst formation rate compared to the control, LiCl, and Klotho + LiCl treatment groups (31.62 ± 2.29 vs. 19.98 ± 1.42, 11.91 ± 1.43, and 16.16 ± 1.26, respectively, P < 0.05). Additionally, the blastocyst formation rate in the LiCl-treated group was significantly lower than that in both the control and KP-treated groups. The KP-treated group also showed the greatest number of cells in blastocysts, compared to the control and LiCl-treated groups (66.71 ± 3.82 vs. 51.36 ± 2.46 and 48.33 ± 2.43, respectively, P < 0.05).

### Transcript and protein expression of Wnt signaling-related factors in porcine oocytes

**Figure 4:**
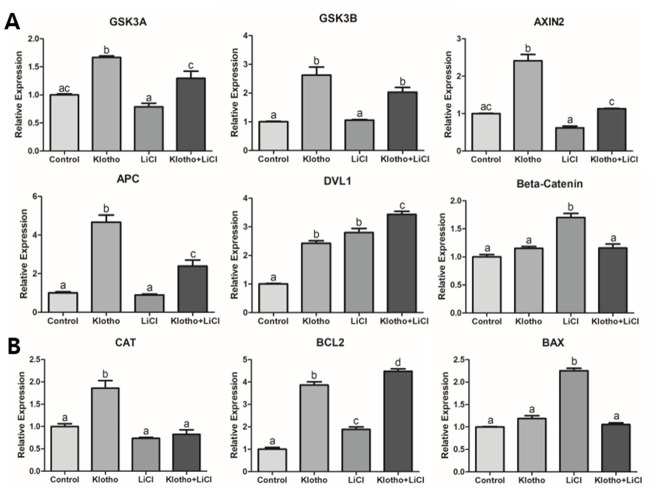
**Relative quantitative mRNA expression through real-time PCR.** The relative expression of mRNA transcripts related to (**A**) Wnt signaling (*GSK3A, GSK3B, AXIN2, APC, DVL1, and β-Catenin*), (**B**) antioxidant activity (*CAT*), and apoptosis (*BAX and BCL2*), are shown for the four different experimental groups: control, Klotho, LiCl, and Klotho + LiCl. The experiments were performed in triplicate, using mature oocytes from five different biological replications. Bars with letters indicate significant differences (*P* < 0.05).

**Figure 5:**
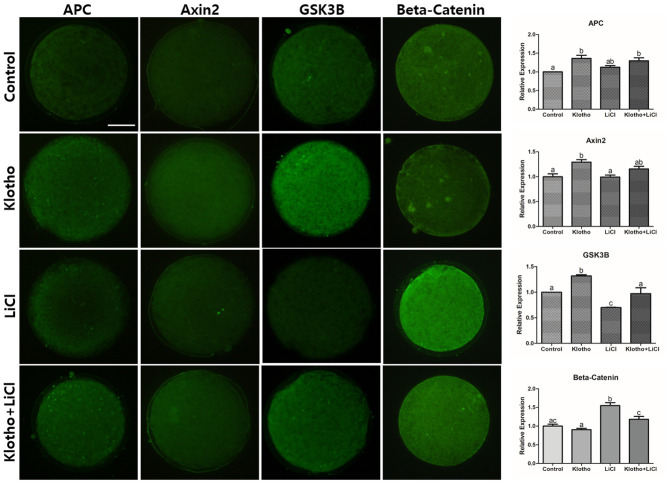
**Immunocytochemistry of Wnt signaling-related proteins (APC, Axin2, GSK3B, and β-Catenin) in mature porcine oocytes.** Fluorescence intensities were measured using ImageJ. Bars with letters indicate significant differences (*P* < 0.05). X400 Magnifications.

We evaluated the expression levels of mRNA transcripts and proteins involved in the Wnt signaling pathway. First, we performed qRT-PCR on denuded oocytes (Figure 4). The KP-treated group showed significant increase in the expression levels of *GSK3A* and *GSK3B* compared to the LiCl-treated and control groups, although no differences were observed between LiCl-treated and the control groups. In contrast, *DVL1* was upregulated in all treatment groups compared to the control group, and interestingly, the KP-treated group also exhibited a sharp increase in *AXIN2* and *APC* expression, compared to the other groups. On the other hand, *β-catenin* showed the highest expression levels in the LiCl-treated group. The KP-treated group also exhibited a significant increase in the expression level of *Catalase (CAT)*. Additionally, *BCL2* was significantly upregulated in all treatment groups compared to the control, but especially in the KP and co-treated groups; while, *BAX* showed no differences among the treatment groups except for the LiCl-treated group (P < 0.05). We then investigated the expression levels of Wnt signaling-related proteins in porcine oocytes using ICC (Figure 5). The expression levels of APC, AXIN2, and GSK3B in the KP-treated group were significantly higher than those in the control group. Interestingly, the trend exhibited by β-Catenin expression levels differed from those exhibited by the other proteins, as the LiCl-treated group showed the highest expression levels among the groups, while the KP-treated group showed no difference in expression levels compared to the control group.

### Antioxidative properties and ATP production in porcine oocytes

**Figure 6:**
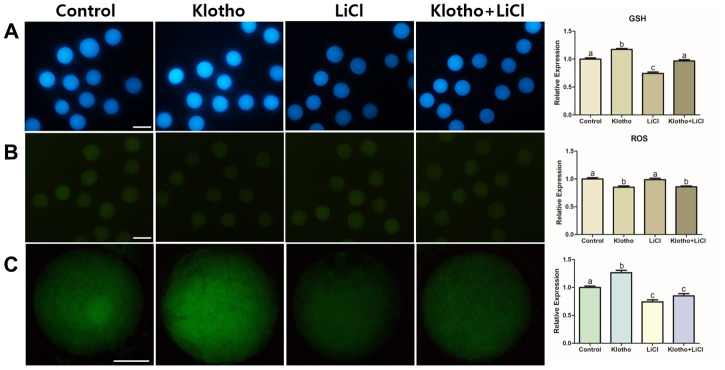
****(**A**) GSH and (**B**) ROS staining performed in mature oocytes. X100 Magnification. (**C**) ATP content assay of mature porcine oocytes from different experimental groups. X400 Magnifications. Bars with letters indicate significant differences (*P* < 0.05).

As Wnt signaling has a close relationship with antioxidant activity, we investigated the GSH and ROS levels in the porcine oocytes. The expression levels of GSH were the highest in the KP-treated group and the lowest in the LiCl-treated group, although no significant difference in GSH expression was observed in the co-treated group compared to that in the control group (Figure 6A). Similarly, the KP- and co-treated groups showed significantly lower ROS expression levels compared with the control and LiCl-treated groups (Figure 6B). Lastly, we evaluated the relative expression levels of ATP stained by BODIPY (Figure 6C). The KP-treated group showed the highest expression of ATP out of the treatment groups, and the relative expression levels of ATP in the LiCl and co-treated groups were significantly lower than that in the control group (P < 0.05).

## DISCUSSION

To our knowledge, this study is the first to investigate the effects of KP and its inhibition of Wnt signaling in porcine oocytes. The results of this study suggest that KP may function in porcine oocyte development by improving the maturation process, enhancing subsequent embryonic development and, increasing antioxidant activity and energy production, thereby increasing the competence of oocytes. Likewise, the results of this study suggest the involvement of Wnt signaling in the action of KP and this effect was examined through using LiCl as an activator of Wnt signaling. Serial concentrations of KP increased the expansion of cumulus cells and embryonic development in the porcine oocytes. These results suggest that the inhibition of Wnt signaling by KP had a significant influence on the porcine oocytes, following our hypothesis.

Several studies have reported that ovarian follicles sized 5-8 mm have greater potential for maturation into oocytes in pigs [21–23]. Our analysis of KP expression in porcine ovaries and follicular fluid showed a similar result [21–23]; follicles of 5-8 mm diameter contain more KP than the follicles sized 2-5 mm. Besides, we confirmed the expression of KP in both ovary and oocytes through IHC, ICC and ELISA. Since there is no information available regarding the concentration range of KP during oocyte or cell treatment, we used the optimal KP concentration suggested by the results of our ELISA analysis. We found that 5 pg/ml KP was the optimal concentration, as it significantly enhanced cumulus cell expansion in porcine COCs as well as their subsequent embryonic development. In keeping with the suggestion that Wnt signaling may be inhibited by activation of Klotho, including regulation of GSK3B [18–20] and its direct influences of Wnt signaling on oocytes [14, 15], we focused our experimental design on the role of Wnt signaling during porcine oocyte maturation. We used LiCl to confirm Wnt signaling activity in the cells, as it is characterized as a specific activator of Wnt signaling [13, 16, 24].

After validating the presence of KP in ovaries and oocytes and optimizing KP and LiCl concentrations for treatment during IVM, we investigated the competence of porcine oocytes by analyzing the relative expression of GDF9 and BMP15, which are known to be representative of oocyte competence [25]. The expression levels of GDF9 and BMP15 were significantly increased in porcine oocytes treated with KP, and decreased in those treated with LiCl. Therefore, our results support the hypothesis that Wnt signaling is inhibited by KP and activated by LiCl. In the ‘canonical’ Wnt signaling pathway, β-Catenin is known to be the main downstream factor of this signaling. The presence of *Wnt* protects *β-Catenin* from degradation and phosphorylation, thus allowing translocation of *β-Catenin* into the nucleus for transcription [26, 27]. This process has been shown to have a negative effect on the competence and development of oocytes and embryos in pigs and mice [13, 14]. In contrast, absence of Wnt protein allows β-Catenin to be degraded by the β-Catenin destruction complex, which is composed of Axin, glycogen synthase kinase 3 (GSK3), adenomatosis polyposis coli (APC), and protein phosphatase 2A (PP2A) [28, 29]. These components are regulated by Frizzled receptors/low-density-lipoprotein-related protein5/6, and the Dishevelled (Dvl) mechanism [30–32]. Therefore, we hypothesized that β-Catenin activity, controlled by Wnt signaling, is fundamental to the maturation process of porcine oocytes.

LiCl is known to activate Wnt signaling. More specifically, it mimics Wnt signaling by inhibiting GSK3B, thereby stabilizing β-Catenin in the cytosol [33, 34]. After treating porcine oocytes with LiCl, we found a significant decrease in the embryonic development and a significant reduction in GSK3B protein expression. This implies that LiCl could influence the Wnt signaling pathway, thereby impairing development and signaling in the porcine embryo. The results of the mRNA and protein expression analyses (Figures 4 and 5) support the interpretation, together with the previous studies [18–20] that the inhibition of Wnt signaling by KP led to activation of β-Catenin destruction complex-related factors (*GSK3A, GSK3B, AXIN2, APC,* and *DVL1*), resulting in *β-Catenin* showing a similar expression level to the control (Figure 4A). This expression pattern was similar to that exhibited by β-Catenin protein (Figure 5). We could thus partially deduce from our results that KP treatment significantly increased porcine oocyte maturation, and this may be caused by Wnt signaling activity. This interpretation is corroborated by the results of the qRT-PCR and ICC analyses, and validated by LiCl treatment.

The functions of Wnt signaling are not limited to embryonic development, cell fate specification and cell proliferation [12, 35, 36], because Wnt signaling also has a relationship with antioxidant properties [37]. Chae et al. proposed that peroxiredoxin1 (PRDX1) knockdown caused an increase in ROS expression during embryogenesis, and PRDX1 has been found to be closely related to Wnt signaling. Therefore, we hypothesized that the expression levels of GSH, including *CAT*, would also exhibit a close relationship with Wnt signaling. In this study, we evaluated intracellular levels of GSH in porcine oocytes, which make it possible to track the antioxidant activity in porcine oocytes. The KP-treated group showed the highest expression levels of GSH (Figure 6A) and significantly decreased expression of ROS. This implies that KP has the antioxidant property in the porcine oocytes, in accordance with the claims of previous studies that KP is improves cellular survival by increasing antioxidant properties [38, 39]. Lastly, we observed strong regulation of *CAT* that has a close relationship with Wnt signaling [40], in the KP-treated group (Figure 4B). It has also been proposed by Costa et al. that Wnt signaling is related to energy production, as confirmed through impairment of mitochondrial function via the endoplasmic reticulum stress [41]. Therefore, we hypothesized that Wnt signaling might increase ATP production in porcine oocytes, and indeed, we found significantly increased expression of ATP in the KP-treated group. Moreover, as the LiCl-treated oocytes showed reduced ATP expression (Figure 6C), our results may be assumed to support the hypothesis that activation of Wnt signaling impairs oocyte competence and maturation by hindering energy production in the cell.

To date, the functions of Klotho are still being revealed in medical and reproductive studies [42–44]. Klotho has been found to have a possible relationship with Wnt signaling [45, 46], including antioxidant properties [47]. In this study, we focused on the relationship between KP and Wnt signaling, and their influence on porcine oocytes. After treating porcine oocytes with KP and verifying the presence of Wnt signaling using LiCl treatment, we found that KP and Wnt signaling intervened significantly with the maturation of the porcine oocytes, consistent with the results of previous studies. A novel aspect of our research is that it may be the first study to identify the presence and concentration of KP in the PFF, the ovarian tissue and in the oocytes, thereby confirming its significance in porcine oocyte maturation via the Wnt signaling pathway. Collectively, we conclude that the KP-Wnt signaling mechanism may be a significant regulator of maturation and development in porcine oocytes. Our results also suggest that treatment with KP can be beneficial to the maturation, embryonic development, and antioxidant properties of porcine oocytes.

## MATERIALS AND METHODS

### Animals and chemicals

Pig ovaries were collected following slaughter. In terms of the research ethics regarding the use of these ovaries, the Institutional Animal Care and Use Committee (IACUC) of Seoul National University carried out ethical screening, then approved the ovaries for research use (no. SNU-171212-2). All chemicals used in this study were purchased from the Sigma-Aldrich Chemical Company, unless otherwise specified.

### Immunohistochemistry (IHC)

The porcine ovaries were collected and fixed in 4% paraformaldehyde. IHC was performed on 3 μm paraffin-embedded tissue sections from the tissue blocks. Histological architecture of the ovary was assessed using Hematoxylin and Eosin (H&E)-stained sections, through an optical microscope (Olympus, BX41, Japan). To detect Klotho antigen, the tissue sections were stained with a rabbit polyclonal Klotho antibody (ab98111, Abcam), followed by Envision+System-HRP Labelled Polymer anti-Rabbit antibodies (K4003; Dako). Image processing was performed with a Leica DMI 6000B microscope, using a DFC350 camera. Expression levels were classified into three categories: ‘mild’, ‘moderate’, and ‘strong’.

### Enzyme-linked immunosorbent assay (ELISA)

Porcine follicular fluid (PFF) from 2-5 mm- and 5-8 mm-sized follicles was collected from porcine ovaries, and used to determine the concentration of KP. ELISA kit for Klotho (MBS9359499, Mybiosource, San Diego, CA, USA) was used and KP concentration was measured according to the manufacturer’s instruction. The concentration was expressed as pg per ml of PFF.

### Oocyte retrieval and *in vitro* maturation

Porcine ovaries from prepubertal gilts were transferred to the laboratory, and COCs were retrieved by slicing with sterilized surgical blades and forceps. The COCs were then washed three times in washing medium comprising 9.5 g/L tissue culture medium-199, 10 mM N-piperazine-N’-[2-ethanesufonic acid] (HEPES), 5 mM sodium hydroxide, 0.3% polyvinyl alcohol (PVA), 2 mM sodium bicarbonate, and 1% penicillin-streptomycin (Invitrogen). COCs containing more than three layers of cumulus cells and homogenous cytoplasm were carefully selected and cultured at 39°C under conditions of 5% CO_2_, in 95% humidified air. The cells were cultured in IVM medium comprising TCM-199, 10 μL/mL insulin-transferrin-selenium solution (ITS-A) 100X (Invitrogen), 0.91 mM sodium pyruvate, 0.57 mM cysteine, 10% PFF (vol/vol), 10 ng/mL epidermal growth factor, 10 IU/mL equine chorionic gonadotropin (eCG), and 10 IU/mL human chorionic gonadotropin (hCG). After 22 h of culture with hormones, the COCs were transferred to hormone-free IVM medium for an additional 20-22 h. For the KP treatment, we used serial standards containing KP from the ELISA kit, as it was impossible to import KP to Korea. KP concentrations were prepared from the standards for the treatments.

### Cumulus cell expansion assessment

Oocytes exhibiting cumulus expansion were evaluated. The degree of cumulus expansion was determined by the morphology of the COCs, following the method described previously [48]. COCs exhibiting no expansion were classified as degree 0, as they were characterized by a complete detachment of cumulus cells (CCs) from oocytes. A classification of degree 1 indicated COCs with spherical and compact CCs that were not expanded, while COCs were classified as degree 2 if only the outermost layers of the CCs were expanded. A classification of degree 3 was likewise given to COCs which showed expansion in all layers of the CCs except for the corona radiata, while COCs which showed full expansion in all layers including the corona radiata were classified as degree 4.

### Parthenogenetic activation (PA)

The process of PA had been described in a previous study [49]. Mature oocytes were denuded with 0.1% hyaluronidase, they were then washed in Tyrode’s albumin lactate pyruvate (TALP) medium. Oocytes exhibiting clear membranes with the first polar bodies and homogeneous cytoplasm were then gradually equilibrated in activation medium comprising 0.5 mM HEPES, 0.28 M mannitol, 0.1 mM MgSO_4_, and 0.1 mM CaCl_2_. The cells were subsequently transferred to a 3.2 mm spaced double electrode chamber filled with the activation medium, and activated by 60 μs electric stimulation with a direct current pulse of 1.5 kV/cm using a BTX Electro-Cell Manipulator 2001 (BTX Inc., San Diego, CA). The activated oocytes were then washed in porcine zygote medium-5 (PZM-5) and transferred to 40 μL droplets of PZM-5, covered with mineral oil, and cultured at 39°C in a humidified atmosphere of 5% CO_2_, 5% O_2_, and 90% N_2_ for 7 days.

### Embryo evaluation and total cell count after PA

The day on which PA was performed and activated oocytes were transferred to the *in vitro* culture (IVC) medium was denoted as Day 0. Embryos exhibiting even cleavage were observed under a stereomicroscope on Day 2 (48 h). On Day 7 (168 h), blastocyst formation was evaluated and total cell numbers counted. Following collection of the blastocysts, they were washed in PBS, and then fixed for 1 h in 4% paraformaldehyde (w/v) in PBS. The blastocysts were then stained with 5 μg/mL of Hoechst 33342 for 5 min. After washing with PBS, the stained blastocysts were mounted on glass slides, covered with cover slips. The total cell numbers of the blastocysts were then counted under a fluorescence microscope (Nikon Corp.) at 400× magnification.

### Immunocytochemistry

Mature oocytes showing the first polar body were selected and washed in PBS, then fixed in 4% paraformaldehyde (w/v) in PBS for at least 1 h. Permeabilization was performed by treating the cells with 1% Triton X-100 (v/v) in distilled water for 1 h at 39°C, washing four times in 1% PVA, and then incubating in 2% BSA for 2 h to block non-specific sites. The oocytes were then transferred directly into 2% BSA containing the primary antibody for Klotho (1:200; ab98111, Abcam), BMP15 (1:200; PA5–34401, Thermo Fisher Scientific), as well as GDF9 (1:200; ab93892, Abcam), APC (1:400; ab72040, Abcam), AXIN2 (1:100; ab109307, Abcam), GSK3B (1:100; ab227208, Abcam), and β-Catenin (1:200; ab16051, Abcam). The oocytes with antibodies were incubated at 4°C overnight. Following incubation, the oocytes were washed four times in 1% PVA droplets, and then incubated with a secondary fluorescein isothiocyanate-conjugated anti-rabbit polyclonal antibody (1:200, ab6717, Abcam. Cambridge, UK) at 37°C for 2 h in darkness. After washing with 1% PVA, the oocytes were then counterstained with 5 μg/mL Hoechst-33342 for 5 min. They were mounted on glass slides with cover glasses, and sealed with transparent manicure. The fluorescence measurements were performed using ImageJ (version 1.46r; National Institute of Health, USA). At least 30 oocytes from each treatment group (Control, KP, LiCl, and KP/LiCl cotreatment) were used for the analysis.

### Analysis of mRNA transcripts expression by relative quantitative real-time PCR

Mature oocytes from treatment groups were stored at -80°C. A total of 400 denuded oocytes from each group were then used for RNA extraction using the RNAqueous^TM^ Micro Kit (Invitrogen, Vilnius, Lithuania). The total RNA yield was measured on a NanoDrop 2000 Spectrophotometer (Thermo Fisher Scientific, Wilmington, DE, USA). The extracted RNA was then immediately used for complementary DNA (cDNA) synthesis using amfiRivert cDNA synthesis Platinum Master Mix 0 (genDEPOT, Houston, TX, USA), according to the manufacturer’s instruction. For the subsequent relative quantitative real-time PCR, 0.4 μL (10 pmol/μL) forward and reverse primer, 10 μL SYBR Premix Ex Taq (Takara, Otsu, Japan), 8.2 μL of Nuclease Free Water, and 1 μL cDNA were added to each reaction mixture, and the samples then moved to a PCR plate (Micro-Amp Optical 96-Well Reaction Plate, Applied Biosystems, Singapore). The mixtures were amplified using the Applied Biosystems StepOneTM Real-Time PCR System (Applied Biosystems, Waltham, MA, USA). Forty cycles of reactions were performed, with the following conditions: 15 s denaturation at 95°C, 1 min annealing at 60°C, and 1 min extension at 72°C. The primer sequences used in this experiment are listed in Table 1. The expression of each target gene was quantified relative to that of the endogenous control gene (*GAPDH*). The relative expression of each gene was calculated using the following equation:

R = 2^-[ΔCt sample - ΔCt control]^.

**Table 1:** Primer sequences used for gene expression analysis.

**Genes**	**Primer sequences (5’- 3’)**	**Product size (bp)**	**Accession No.**
*Klotho*	F: GCTACAGCATCAGACGTGGA	147	XM_021065566.1
	R: TCCCTTCTAGGGGCTGATTT		
*GSK3A*	F: GGTTCAAGAACCGAGAGCTG	118	NM_001315708.1
	R: CTCAGGCACATACTCCAGCA		
*GSK3B*	F: CGAGACACACCTGCACTCTT	200	NM_001128443.1
	R: GTGGAATTGGAAGCTGACGC		
*AXIN2*	F: TGGCATCAAGAAGCAGCAGA	81	XM_021066736.1
	R: GCATTGTCCTCCATCACCGA		
*APC*	F: CCGCAGCTTTAAGGAATCTG	111	NM_001206430.1
	R: GGGCTTTTTGTTTCCTGACA		
*DVL1*	F: CCAGCAGAGTGAAGGAAGCA	86	XM_003127500.6
	R: TCTCCTTCTCTCGACCCAGG		
*BETA-CATENIN*	F: AACCTGCCATCTGTGCTCTC	87	NM_214367.1
	R: GTCCGTAGTGAAGGCGAACA		
*CAT*	F: AGGGAGAGGCGGTTTATTGC	117	NM_001206359
	R: GGACTCGTTGGTGAAGCTCA		
*BCL2*	F: AATGTCTCAGAGCAACCGGG	193	NM_214285
	R: GGGGCCTCAGTTCTGTTCTC		
*BAX*	F: CATGAAGACAGGGGCCCTTT	181	XM_003127290
	R: CATCCTCTGCAGCTCCATGT		

* F, Forward primer; R, Reverse Primer.

### Measurement of intracellular GSH and ROS levels

More than 60 oocytes in total were used in three independent replicates. After 44 h of IVM, the intracellular ROS and GSH levels of oocytes were evaluated by staining with H2DCFDA (2’, 7’-dichlorodihydrofluorescein diacetate; Invitrogen) and CellTracker Blue (4-chloromethyl- 6.8- difluoro- 7- hydroxycoumarin;CMF2HC; Invitrogen), respectively. After gentle denudation, mature oocytes were selected from each group and stained in dark conditions for 30 min in TALP medium containing 10 μM of H2DCFDA or 10 μM of CellTracker Blue. The stained oocytes were then washed four times in TALP medium droplets, and more than ten stained oocytes were transferred into a 4-μl droplet of TALP medium and covered with mineral oil. The intensity of fluorescence was then measured immediately under an epifluorescence microscope (TE2000-S; Nikon, Tokyo, Japan) with UV filters (370 nm for GSH and 460 nm for ROS). The fluorescence intensities of the oocytes were analyzed using ImageJ software. For the comparison, the intensity of the control group was arbitrarily normalized to ‘1-fold’.

### ATP content assay

Mature oocytes were denuded and washed three times in 1% PVA droplets, then fixed in 4% PFA for 1 h at room temperature. The fixed oocytes were then washed again with 1% PVA several times, and transferred to PBS containing 0.5 μM BODIPY FL ATP (BODIPY-ATP; A12410; Molecular Probes, Eugene, OR, USA), in which they were incubated for 1 h at room temperature, avoiding light. The stained oocytes were then washed in 1% PVA in PBS, and then they were mounted on glass slides, covered with slips, and sealed with transparent manicure. An epifluorescence microscope (TE2000-S; Nikon) was used for capturing images. The ATP content intensities were measured using ImageJ software. The intensities of the control group were arbitrarily standardized as 1-fold.

### Statistical analysis

All experiments in this study were performed in triplicates at least. Statistical analysis was performed using GraphPad PRISM 5.01 (PRISM 5, GraphPad Software, Inc., San Diego, CA 92108, USA). To determine significant differences between experimental groups, all data were expressed in terms of mean ± S.E.M, and analyzed using a one-way analysis of variance (ANOVA) with Tukey’s Multiple Comparison Test. P values < 0.05 represented significant differences.
